# Supplementary material for: Asymmetric Synthesis of Quaternary Hydantoins via a Palladium-Catalyzed Aza-Heck Cyclization
Source: J Am Chem Soc. 2025 Nov 14;147(49):44692–8. doi: 10.1021/jacs.5c16022 (PMC12703750; doi:10.1021/jacs.5c16022)
Supplement: Supplementary file 2 [file ja5c16022_si_002.zip › All NMR FID Files/S1/S1_ProtonNMR/2/pdata/1/email_EM04-060-1_2_1.pdf]

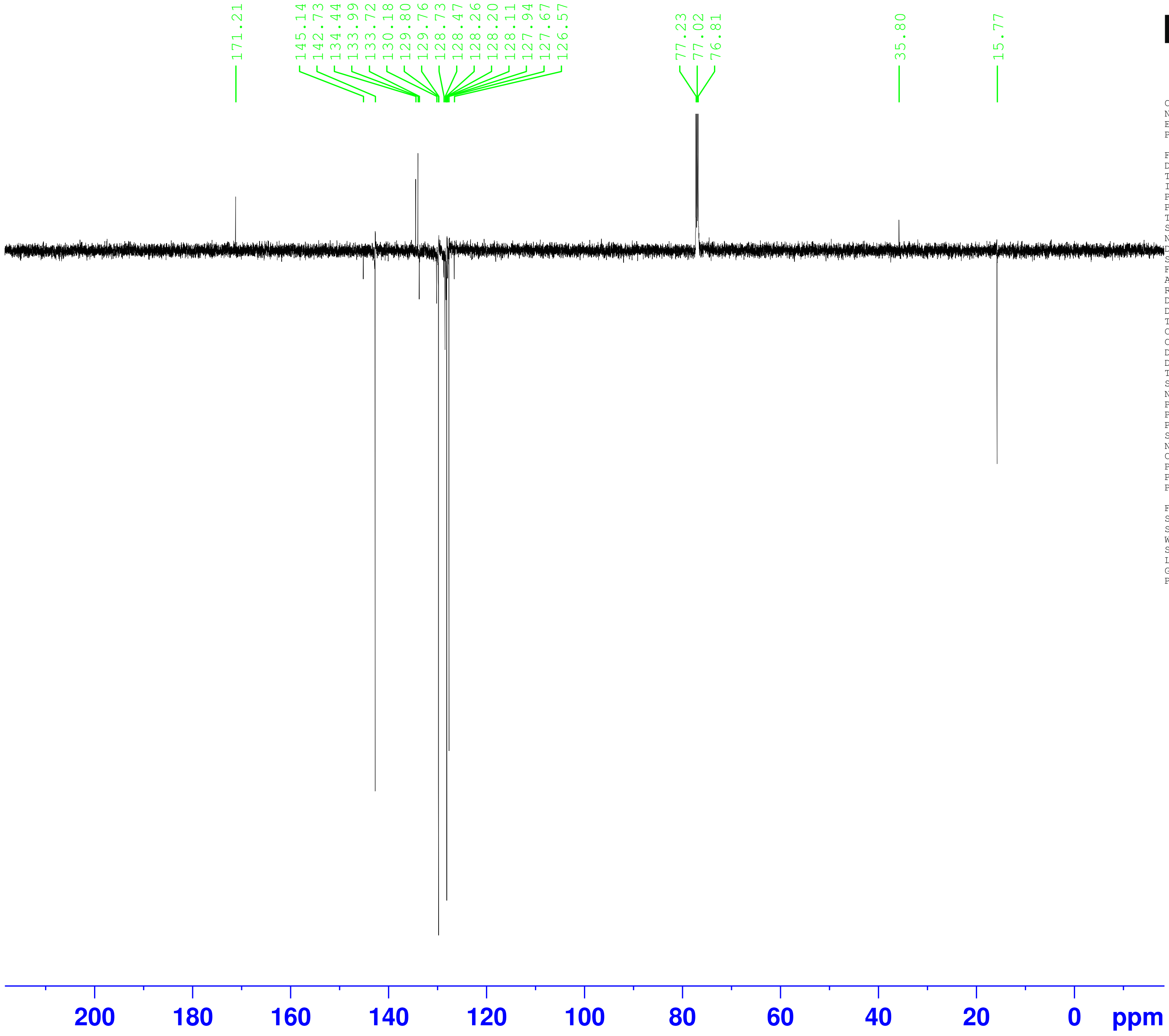


```
Current Data Parameters
NAME             EM04-060-1
EXPNO                     2
PROCNO                    1

F2 - Acquisition Parameters
Date_              20230419
Time                  18.54 h
INSTRUM   Avance Neo 600 at University of Delaware
PROBHD    Z185170_0001 (
PULPROG                jmod
TD                    65536
SOLVENT               CDCl3
NS                     1024
DS                        4
SWH               35714.286 Hz
FIDRES             1.089913 Hz
AQ                0.9175040 sec
RG                      101
DW                   14.000 usec
DE                    19.25 usec
TE                    298.0 K
CNST2           145.0000000
CNST11            1.0000000
D1               2.00000000 sec
D20              0.00689655 sec
TD0                       1
SFO1            150.9178988 MHz
NUC1                    13C
P1                    11.78 usec
P2                    23.56 usec
PLW1           106.00000000 W
SFO2            600.1324005 MHz
NUC2                     1H
CPDPRG[2            waltz65
PCPD2                 70.00 usec
PLW2             6.90000010 W
PLW12            0.09034800 W

F2 - Processing parameters
SI                    32768
SF              150.9028085 MHz
WDW                      EM
SSB                       0
LB                     1.00 Hz
GB                        0
PC                     1.40
```
